# A Novel Bacteriophage with the Potential to Inhibit *Fusobacterium nucleatum*-Induced Proliferation of Colorectal Cancer Cells

**DOI:** 10.3390/antibiotics14010045

**Published:** 2025-01-07

**Authors:** Ho Yin Pekkle Lam, Meng-Jiun Lai, Pin-Chun Wang, Wen-Jui Wu, Li-Kuang Chen, Hsiang-Wei Fan, Chun-Chieh Tseng, Shih-Yi Peng, Kai-Chih Chang

**Affiliations:** 1 Department of Biochemistry, School of Medicine, Tzu Chi University, Hualien 970374, Taiwan; pekklelavabo@mail.tcu.edu.tw; 2 Institute of Medical Science, College of Medicine, Tzu Chi University, Hualien 970374, Taiwan; likuangchen@gmail.com; 3 Department of Laboratory Medicine and Biotechnology, Tzu Chi University, Hualien 970374, Taiwan; monjou@mail.tcu.edu.tw (M.-J.L.); 110323102@gms.tcu.edu.tw (P.-C.W.); w200811@mail.tcu.edu.tw (W.-J.W.); 4 Branch of Clinical Pathology, Department of Laboratory Medicine, Buddhist Tzu Chi General Hospital, Hualien 970473, Taiwan; 5 Master Program in Biomedical Science, School of Medicine, Tzu Chi University, Hualien 970374, Taiwan; 112333108@gms.tcu.edu.tw; 6 Department and Graduate Institute of Public Health, Tzu Chi University, Hualien 970374, Taiwan; tsengcc@mail.tcu.edu.tw; 7 Department of Laboratory Medicine, Hualien Tzu Chi Hospital, Buddhist Tzu Chi Medical Foundation, Hualien 970473, Taiwan

**Keywords:** *Fusobacterium nucleatum*, colorectal cancer, antibiotic resistance, bacteriophage, phage therapy

## Abstract

Background: Increasing evidence shows that *Fusobacterium nucleatum* (*F. nucleatum*) largely affects colorectal cancer (CRC) growth and progression; therefore, the inhibition of intratumoral *F. nucleatum* may be one realistic approach to combat CRC. Although antibiotics are helpful in eliminating bacteria, the major problem remains the rise of potential antibiotic-resistant strains and antibiotic-associated adverse effects. Currently, bacteriophage therapy has gained interest because of its high selectivity to bacterial hosts and may become a realistic approach in treating bacteria-associated cancers. Methods: In this study, a new *F. nucleatum* bacteriophage, ØTCUFN3, was isolated and its biological characteristics were identified. In vitro and in vivo studies were performed to investigate the effect of ØTCUFN3 in combating *F. nucleatum*-induced CRC growth. Results: By applying ØTCUFN3 to *F. nucleatum*-induced CRC cell lines, p53^+/+^, and p53^−/−^ isogenic HCT116 cells, our results revealed an inhibition of CRC proliferation and the expression of epithelial-to-mesenchymal transition (EMT) markers. ØTCUFN3 injection also reduced the growth of *F. nucleatum*-induced mouse xenografts. Conclusions: Our results demonstrated the use of *F. nucleatum* bacteriophage against CRC, laying the foundation for the future usage of bacteriophage in cancer treatment.

## 1. Introduction

*Fusobacterium nucleatum* (*F. nucleatum*) is an anaerobic oral commensal associated with periodontal diseases, gastrointestinal diseases, and colorectal cancers (CRC) [1]. Increasing evidence showed that intestinal *F. nucleatum* is higher in CRC patients than in healthy individuals [2]. The translocation of *F. nucleatum* from the mouth to CRC was shown to happen through the bloodstream route [3] or the alimentary tract route [4]. D-galactose-β(1–3)-*N*-acetyl-d-galactosamine (Gal-Gal-NAc), a carbohydrate that is overexpressed in CRC, has been shown to be an essential factor that is recognized by fibroblast activation protein-2 (Fap2), a surface protein of *F. nucleatum* [5]. Therefore, successful colonization of *F. nucleatum* on CRC is seen to be positively correlated with Gal-GalNAc expression [5].

The colonization of *F. nucleatum* on CRC has been suggested to accelerate CRC proliferation [6] and promote invasion and metastasis [5]. The enhancement of CRC’s stemness may also lead to resistance to chemotherapy and tumor recurrence [7]. In addition, *F. nucleatum* predominantly interacts with the tumor microenvironment, leading to a pro-inflammatory environment [8], macrophage polarization [9,10], and the suppression of T cells [11] and NK cells [12].

Because *F. nucleatum* largely affects CRC growth and progression, the inhibition of intratumoral *F. nucleatum* may be a potential way to combat CRC. The use of the antibiotic metronidazole has significantly reduced intratumoral *F. nucleatum* load and tumor growth in mouse xenografts [13], suggesting that the effective removal of *F. nucleatum* may reduce CRC progression. Nevertheless, the major challenge with antibiotic treatment remains the emergence of resistant strains [14] and its adverse drug effects [15].

Bacteriophage therapy has gained rising interest because of its high selectivity to bacterial hosts. Although several *F. nucleatum* bacteriophages have been found [16,17,18], none have been tested on targeting *Fusobacterium*-induced CRC growth. In this study, a new *F. nucleatum* bacteriophage, ØTCUFN3, was identified. The application of ØTCUFN3 to *F. nucleatum*-induced CRC cell lines revealed the suppression of CRC cell growth and the expression of epithelial-to-mesenchymal transition (EMT) markers. ØTCUFN3 injection also reduced the growth of mouse xenografts. This reduction was accompanied by an infiltration of inflammatory cells into the xenografts, suggesting that ØTCUFN3 may also stimulate the host immunity to combat cancers.

## 2. Results

### 2.1. Isolation and Morphologic Characteristics of ØTCUFN3

**Figure 1:**
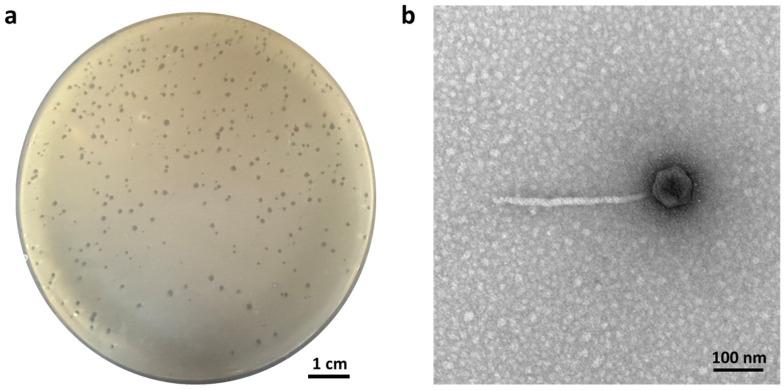
Plaquing phenotype and morphology of ØTCUFN3. (**a**) Representative images of lytic plaques of ØTCUFN3 against *F. nucleatum* strain 34597 on a double-layer agar plate. (**b**) Representative transmission electron microscopic (TEM) image of ØTCUFN3, showing an icosahedron head with a noncontractile long tail attached to it.

By screening sewage samples through a mixture of 33 *F. nucleatum* strains, one bacteriophage with the ability to form plaque on *F. nucleatum* lawns was found (Figure 1a) and was named ØTCUFN3. Morphological analysis of ØTCUFN3 was performed by TEM and was categorized as a member of the family Siphoviridae [19]. This bacteriophage includes an icosahedron head of about 93 ± 3 nm and a non-contractile tail of 307 ± 10 nm long and 13 ± 1 nm wide (Figure 1b).

### 2.2. Genome Analysis of ØTCUFN3

**Figure 2:**
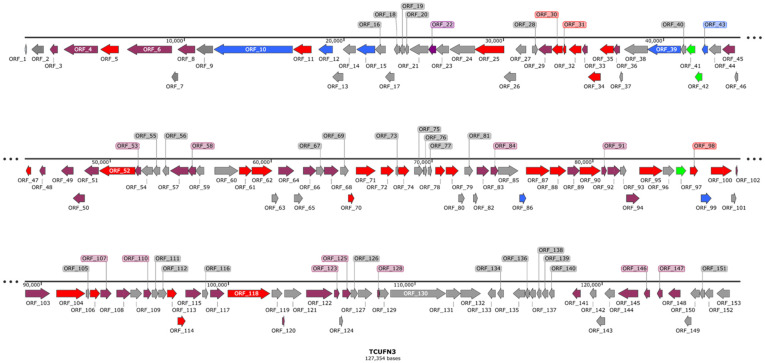
Genomic organization of ØTCUFN3. The bacteriophage contains 127,354 bp and 153 open reading frames (ORFs). ORFs predicted as hypothetical proteins are marked as grey; structural proteins as blue; DNA- and RNA-related proteins as red; endolysin-related proteins as green; unclassified functional proteins as purple. The figure was created using the SnapGene program, http://www.snapgene.com (accessed on 16 October 2024).

Genome sequencing of ØTCUFN3 revealed a genome size of 127,354 bp with a GC content of 26% and 153 predicted open reading frames (ORFs). Of the 153 predicted ORFs, 72 were predicted as hypothetical proteins, whereas 81 were predicted as functional proteins, including 7 structural proteins, 29 DNA- and RNA-related proteins, 3 endolysin-related proteins, and 42 unclassified functional proteins (Figure 2; Table S1). On the other hand, ØTCUFN3 revealed a high degree of sequence similarity to the published *Fusobacterium* bacteriophage Fnu1 (NCBI accession number: NC_055035; sequence similarity: 89.89%) [18], FnuS_FNU2 (NCBI accession number: OQ808963; sequence similarity: 89.88%), FnuS_FNU3 (NCBI accession number: OQ808965; sequence similarity: 90.30%), and phiKSUM (NCBI accession number: OR492276; sequence similarity: 83.47%). Since FnuS_FNU2 and FnuS_FNU3 genomes are highly identical to Fnu1 (sequence similarity: 99%), we only compared the genome of ØTCUFN3 with that of Fnu1 and phiKSUM. Genome alignment using Mauve revealed that 132 out of 153 predicted ORFs from ØTCUFN3 (Figure S1a) were homologs to the predicted proteins in Fnu1 (Figure S1b), and 78 out of 153 showed hits to the predicted proteins of phiKSUM (Figure S1c). When comparing to the structural proteins, both the portal protein (TCUFN3_24) and terminase (TCUFN3_25) in ØTCUFN3 exhibited high identities (>90%) to their homologs in Fnu1 (gp015 and gp017, respectively; Figure S1d) but less than 60% identity to those in phiKSUM (CDS0086 and CDS0087, respectively; Figure S1e).

### 2.3. Biological Characteristics of ØTCUFN3

We next identified the biological characteristics of ØTCUFN3. Host range analysis revealed that ØTCUFN3 could inhibit the growth of 11 out of 33 *F. nucleatum* strains in the spot test method and grow as plaques on two out of 33 strains in the double-layer agar method (Table 1). From these results, *F. nucleatum* 34597 was chosen as the host for ØTCUFN3 for further study.

**Figure 3:**
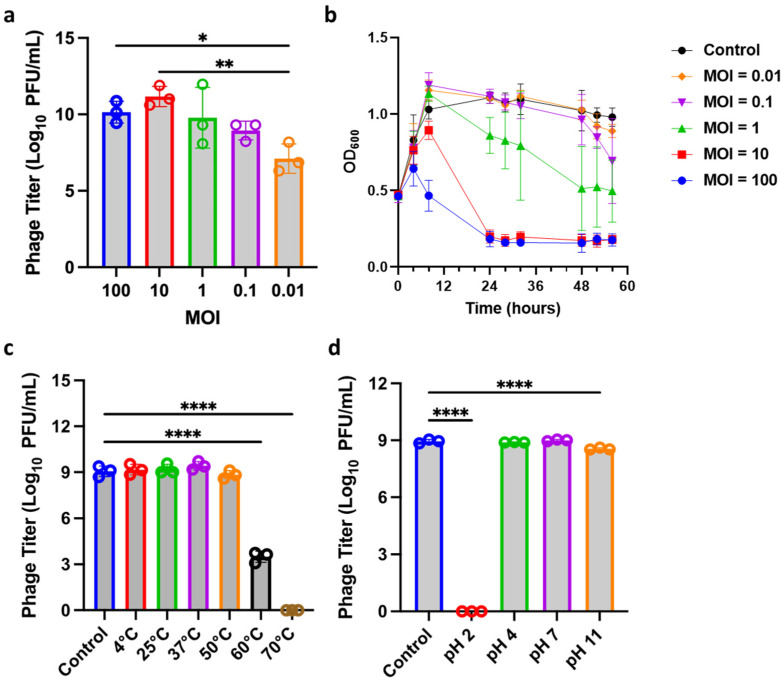
Biological characteristics of ØTCUFN3. (**a**) The optimal multiplicity of infection (MOI) of ØTCUFN3 was determined by infecting *F. nucleatum* 34597. MOI = 10 was found to be the optimal MOI of ØTCUFN3. (**b**) Infection assay of ØTCUFN3 against *F. nucleatum* 34597. MOI = 10 and 100 show complete inhibition after 24 h. (**c**,**d**) Stability of ØTCUFN3 at different (**c**) temperatures and (**d**) pH. Phage incubated in BHI broth at 37 °C was served as a control. Results shown are mean ± S.D. from three independent tests. * *p*-value < 0.005; ** *p*-value < 0.001; **** *p*-value < 0.0001 according to one-way ANOVA.

In order to investigate the optimal multiplicity of infection (MOI), ØTCUFN3 was evaluated against its host from MOI 0.01 to MOI 100. Our results showed that ØTCUFN3 has the highest titer (about 1 × 10^11^ PFU/mL) at MOI = 10, indicating its optimal MOI (Figure 3a). The effect of the lytic ability of ØTCUFN3 was then tested through infecting ØTCUFN3 with *F. nucleatum* 34597 at different MOIs. The results showed that at MOI = 10 or 100, bacterial inhibition could be achieved within the first 24 h, and the bacteria were continuously inhibited even after 48 h. This inhibitory effect decreased in a dose-dependent trend (Figure 3b).

While we observed a successful phage neutralization to their host, we next investigated the effect of temperature and pH on phage stability. ØTCUFN3 was exposed to different temperatures and pH, and the results showed that only at high temperatures, such as 60 °C or 70 °C, did the phage titer drop significantly (Figure 3c). On the other hand, phage stability significantly dropped in extreme pH environments, including pH 2 and pH 11.

### 2.4. ØTCUFN3 Inhibits F. nucleatum-Induced Proliferation and Epithelial-to-Mesenchymal Transition (EMT) of Colorectal Cancer Cells

While *F. nucleatum* has long been suggested to induce the proliferation of CRC cells and tumor development [6], the ability of ØTCUFN3 to suppress *F. nucleatum*-induced colorectal cancer cell growth was investigated using HCT-116 p53 wild-type (p53^+/+^) and p53 knockout (p53^−/−^) colorectal cancer cell lines.

**Figure 4:**
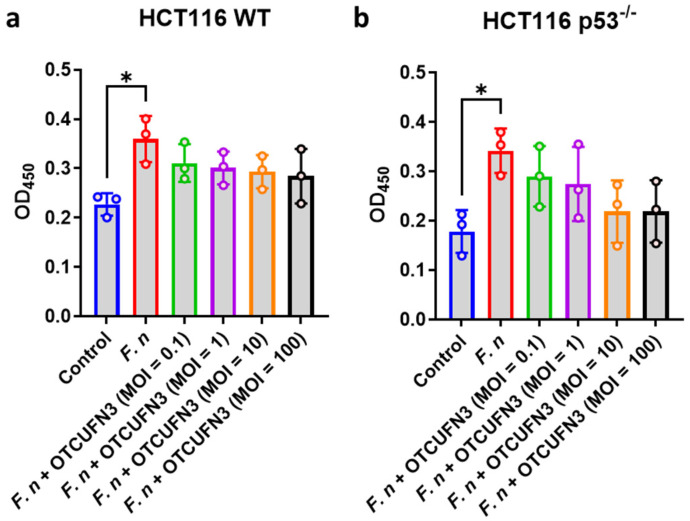
ØTCUFN3 suppresses proliferation of *Fusobacterium nucleatum*-induced p53^−/−^ HCT-116 cells. (**a**) p53 wild-type (p53^+/+^) and (**b**) p53 knockout (p53^−/−^) HCT-116 cells were treated with *F. nucleatum* (F. n.) at an MOI of 100, followed by treatment of ØTCUFN3 at MOIs that ranged from 0.1 to 100 against the bacteria. The cells were cultured for 24 h, and proliferation was measured by CCK-8 assay at a wavelength of 450 nm. Results shown are mean ± S.D. from three independent tests. * *p*-value < 0.05 determined by one-way ANOVA.

The optimal MOI of *F. nucleatum* to infect the cells was determined using a cell counting kit-8 (CCK-8) assay, showing that the best MOI to induce HCT-116 cell proliferation was MOI 100 (Figure S2a,b). As expected, ØTCUFN3 did not induce any changes to HCT-116 cells (Figure S2c,d). To this end, *F. nucleatum* was infected in the cells at MOI 100 (against cells) and treated with different MOIs (against bacteria) of ØTCUFN3. ØTCUFN3 did not inhibit *F. nucleatum*-induced proliferation in p53^+/+^ HCT-116 cells (Figure 4a). However, a slight inhibitory effect was observed in p53^−/−^ HCT-116 cells, although statistical significance was not achieved (Figure 4b).

**Figure 5:**
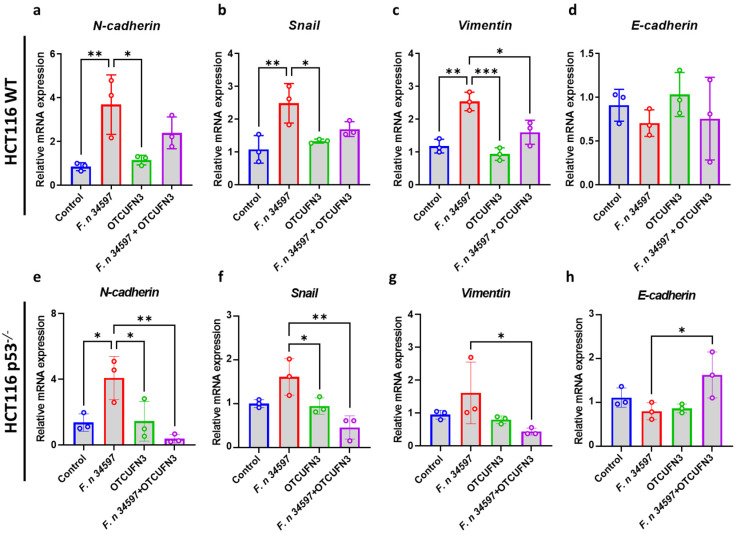
ØTCUFN3 potentially suppresses epithelial-to-mesenchymal transition (EMT) of *Fusobacterium nucleatum*-induced, p53-mutated HCT-116 cells. (**a**–**d**) p53 wild-type (p53^+/+^) and (**e**–**h**) p53 knockout (p53^−/−^) HCT-116 cells were treated with *F. nucleatum* (F. n.) at a MOI = 100 and ØTCUFN3 at a MOI = 10 against bacteria for 24 h. Gene expression of EMT markers, including N-cadherin (**a**,**e**), Snail (**b**,**f**), Vimentin (**c**,**g**), and E-cadherin (**d**,**h**) was measured by qRT-PCR. Results shown are mean ± S.D. from three independent tests. * *p*-value < 0.05, ** *p*-value < 0.01, and *** *p*-value < 0.001 according to one-way ANOVA.

The epithelial-to-mesenchymal transition (EMT) was investigated next, as it is highly associated with the progression of cancer cells [20]. Although not always statistically significant, *F. nucleatum* induces the upregulation of mesenchymal transition, as indicated by increased *N-cadherin*, *Snail*, and *Vimentin* expression in both p53^+/+^ and p53^−/−^ HCT-116 cells. Regarding ØTCUFN3 treatment, ØTCUFN3 treatment only suppressed *F. nucleatum*-induced *Vimentin* expression in p53^+/+^ HCT-116 cells (Figure 5a–c), whereas it significantly downregulated the expression of all the investigated mesenchymal markers, including *N-cadherin*, *Snail*, and *Vimentin* in p53^−/−^ HCT-116 cells (Figure 5e–g). On the other hand, *F. nucleatum* did not affect the epithelial marker, *E-cadherin*, expression in p53^+/+^ HCT-116 cells (Figure 5d,h); but, in p53^−/−^ HCT-116 cells, ØTCUFN3 did upregulate the expression of *E-cadherin* (Figure 5h). Notably, the sole treatment of ØTCUFN3 to the cells did not affect EMT expression compared to the control group. These results, therefore, suggested a potential role of ØTCUFN3 in suppressing *F. nucleatum*-induced, p53-mutated colorectal cancer cell growth and progression.

### 2.5. ØTCUFN3 Inhibits F. nucleatum-Induced Formation of Xenograft Tumors in Mice

**Figure 6:**
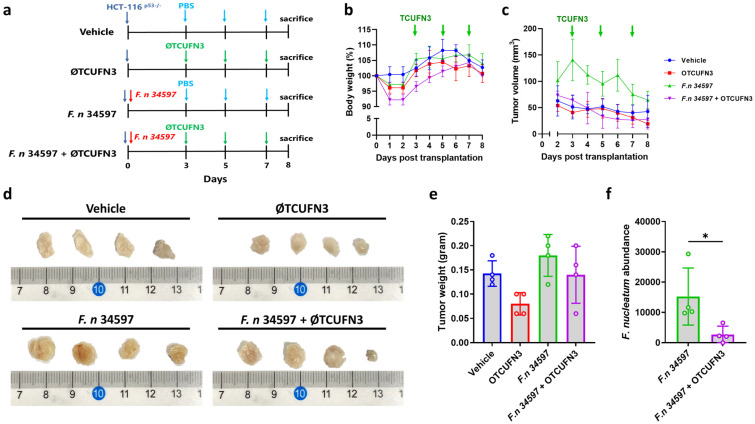
ØTCUFN3 inhibited tumor growth in the HCT-116 p53^−/−^ murine xenograft model. (**a**) Experimental scheme. Briefly, 1 × 10^6^ CFU *F. nucleatum* was mixed with 1 × 10^7^ suspended cells and injected subcutaneously into the right flanks of the mice. 1 × 10^7^ PFU ØTCHFN3 was injected next to the xenograft at days 3, 5, and 7. Mice were sacrificed on day 8. (**b**) Body weight of the mice. (**c**) Tumor volume. For (**b**,**c**), the blue arrow represents the day of ØTCHFN3 injection. (**d**) Photograph of the excised xenograft tumor. (**e**) Tumor weight. (**f**) Abundance of *F. nucleatum* in the tumor. Results shown are mean ± S.D. with four mice in each group.* *p*-value < 0.05 according to one-way ANOVA.

Finally, an attempt was made to observe the therapeutic effect of ØTCUFN3 in the *F. nucleatum*-induced formation of xenograft tumors in mice. p53^−/−^ HCT-116 cells, mixed with or without *F. nucleatum*, were subcutaneously injected into the right flanks of the mice. ØTCUFN3 was injected next to the xenograft tumor at days 3, 5, and 7 post-xenograft injection (Figure 6a). During the experiment, the body weight of the mice did not change (Figure 6b), but *F. nucleatum* infection induced a larger xenograft tumor compared to the non-infected groups, as observed on day two post-injection (Figure 6c). The injection of ØTCUFN3 suppressed the growth of cells infected with *F. nucleatum* (Figure 6c). The tumors were smaller in size and weight in mice receiving ØTCUFN3 treatment than those without ØTCUFN3 treatment (Figure 6d,e). Interestingly, mice injected with non-infected cells also reduced tumor size and tumor weight after treatment with ØTCUFN3 (Figure 6c–e). The tumors were also analyzed for the abundance of *F. nucleatum*. The results suggested that ØTCUFN3 treatment indeed significantly decreased intratumoral *F. nucleatum* (Figure 6f).

**Figure 7:**
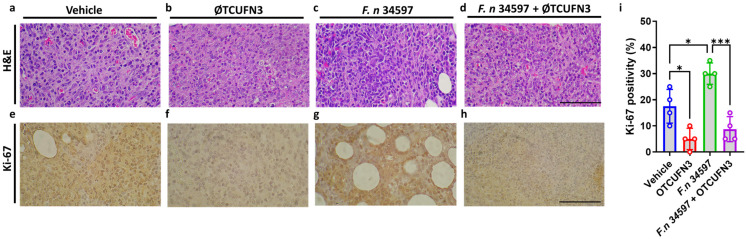
ØTCUFN3 induces tumor necrosis in the HCT-116 p53^−/−^ murine xenograft model. (**a**–**d**) Representative image of H&E-stained sections. ØTCUFN3 treatment induces inflammatory infiltration and significant necrosis in xenografts infected or uninfected with *F. nucleatum*. (**e**–**h**) Representative image of Ki-67-stained sections. (**i**) Percentage of Ki-67 positivity. Scale bar, 100 μm. Results shown are mean ± S.D. with four mice in each group. * *p* < 0.05 and *** *p* < 0.001 according to one-way ANOVA.

Histological analysis of the xenograft tumors revealed that *F. nucleatum* induced higher cell proliferation, as denser cellular content was observed (Figure 7c). On the other hand, ØTCUFN3 treatment induced inflammatory cell infiltration and necrosis, regardless of whether the cells were infected with *F. nucleatum* (Figure 7b,d). This result may provide an explanation for the tumor size reduction by ØTCUFN3 (Figure 6e). An immunohistochemistry staining was then performed to investigate the proliferation marker Ki-67 in the xenograft tissue. As expected, Ki-67 positivity was higher in the *F. nucleatum*-treated xenograft than in the vehicle-treated xenograft (Figure 7e,g,i). ØTCUFN3 treatment reduced the staining intensity of Ki-67 in the *F. nucleatum*-treated xenograft (Figure 7h). However, ØTCUFN3-treated cells also showed a significant reduction in proliferation (Figure 7f,i).

### 2.6. ØTCUFN3 Induces Immune Activation but Does Not Cause Pathological Damage in Healthy Mice

**Figure 8:**
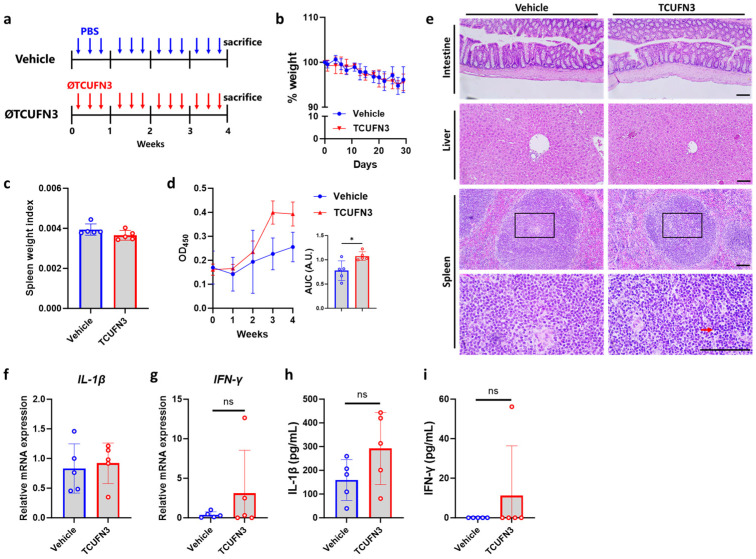
ØTCUFN3 activates immune responses but does not induce pathological injuries. (**a**) Experimental scheme. Vehicle (PBS) or ØTCUFN3 was injected intraperitoneally three times a week into healthy BALB/c mice for four weeks. (**b**) Body weight of the mice. (**c**) Spleen weight index. The calculation was performed by dividing the spleen weight by the body weight. (**d**) Total ØTCUFN3-specific IgG levels in serum every week. Comparison between the two groups was carried out by area under the curve (AUC). (**e**) Representative histology of the intestine, liver, and spleen. Immune activation is indicated by the red arrow. The black bracket highlights the magnified section (400× magnification). Scale bar, 100 μm. (**f**,**g**) Splenic gene expression of IL-1β (**f**) and IFN-γ (**g**). (**h**,**i**) Serum levels of IL-1β (**h**) and IFN-γ (**i**). Results shown are mean ± S.D. with five mice in each group. * *p* < 0.05 according to *t*-test.

As we observed an immune-inducing effect of ØTCUFN3 in a CRC transplant, we wanted to investigate whether ØTCUFN3 exhibited any immune-regulatory or adverse effects in healthy mice. The injection of ØTCUFN3 was performed three times a week into healthy mice for four weeks (Figure 8a). The injection of ØTCUFN3 did not affect the mice’s body weight (Figure 8b) or their splenic weight (Figure 8c). Yet, when investigating their systematic immune response, we found that mice immunized with ØTCUFN3 showed a significantly higher ØTCUFN3-specific IgG response (Figure 8d). Histopathological analysis was carried out on the intestine, liver, and spleen. While no pathological response was observed in the intestine or liver, a slight immune activation was observed in the spleen (Figure 8e). Finally, an attempt was made to investigate the inflammatory response in the mice. We found that the splenic mRNA expression of *IL-1β* and *IFN-γ* was unchanged (Figure 8f–g). Serum IL-1β levels were only slightly, but without statistical significance, increased compared to the control (Figure 8h), whereas serum IFN-γ was not changed (Figure 8i). These results suggested that the injection of ØTCUFN3 did activate, at least in part, the host immune response but did not induce adverse effects.

## 3. Discussion

*F. nucleatum* has long been suggested as one of the important factors in accelerating CRC formation and progression [6,7]. Therefore, removing intratumoral *F. nucleatum* may provide a new strategy for combating CRC. While antibiotics have helped eliminate intratumoral bacteria, the adverse effects are also becoming apparent [19]. In this study, a new *F. nucleatum* bacteriophage was identified, showing the effective suppression of *F. nucleatum*-induced CRC cell growth, which was observed in both in vitro and in vivo experiments.

The tumor microenvironment (TME) is essential in promoting cancer survival and development. The TME contains numerous cytokines secreted from the tumor and tumor-infiltrating lymphocytes [20]. More recent studies showed that intratumoral microbiota may also contribute to the regulation of TME. For example, *F. nucleatum* has been shown to be inversely associated with anti-cancer T-cell response, leading to CRC formation [21]. *F. nucleatum* also produces an inflammatory response, contributing to CRC [22]. Currently, five subspecies of *F. nucleatum* have been identified: *animalis*, *fusiforme*, *nucleatum*, *polymorphum*, and *vincentii.* In this study, we employed *F. nucleatum* 34597, which belongs to the subspecies *animalis*. Although *F. nucleatum* subsp. *animalis* was the most common *F. nucleatum* subspecies associated with CRC [23,24,25], other *F. nucleatum* subspecies have also been recognized in CRC specimens [25], and each of them has been shown to differently affect the TME of CRC [8,23]. However, the possible role of different *F. nucleatum* subspecies in CRC remains poorly understood. Furthermore, a recent study has suggested that *F. nucleatum* subsp. *animalis* is composed of two clades, namely Fna C1 and Fna C2. Fna C1 is predominantly confined to the oral cavity, whereas Fna C2 predominates in the CRC niche [26]. In addition, only Fna C2 is able to induce carcinogenesis [26]. Therefore, a comprehensive investigation of the relationship between *Fusobacterium* and CRC is necessary before fully utilizing phage therapy in cancer treatment. This also leads to another point: a cocktail of *F. nucleatum* phage may be required for a treatment to be successful.

CRC frequently harbors p53 mutations, leading to worsening clinical outcomes [27]. Although a previous investigation identified several molecular events, such as CpG island methylation, microsatellite instability, and CHD7/8 mutation, that can enrich *F. nucleatum* colonization on CRC cells, p53 mutation was not identified to be associated with *F. nucleatum* colonization [28]. This therefore provided us with a reason why infecting *F. nucleatum* with p53^+/+^ and p53^−/−^ HCT-116 cells resulted in similar cell proliferation rates (Figure 4). On the other hand, ØTCUFN3 treatment demonstrated a more substantial outcome in p53^−/−^ HCT-116 cells compared to the wild-type cells (Figure 4 and Figure 5). p53 is essential for cancer suppression; therefore, it is reasonable that the lack of p53 would increase resistance to therapies [29]. Yet, our results revealed that ØTCUFN3 treatment provided a better therapeutic outcome in p53^−/−^ HCT-116 cells than in wild-type HCT-116 cells. Currently, we are unable to explain this phenomenon; however, similar to many drugs that target mutant p53 in cancers [30], future investigation into the therapeutic modulation and molecular mechanism of ØTCUFN3 treatment may help resolve this question.

Bacteriophages are naturally occurring viruses that target bacteria of interest precisely without targeting other bacteria or host cells. However, injecting ØTCUFN3 into the xenograft tumor showed significant inflammatory infiltration and necrosis (Figure 6), which was unexpected. Bacteriophages have been demonstrated to stimulate non-specific innate and adaptive immunity [31] and produce bacteriophage-specific immunoglobins [32,33]. Although these immune responses are attributed solely to the bacteriophages, we cannot exclude the possibility that they may also be harmful to the cells, similar to when immune cells eliminate intracellular pathogens. It was also reported that bacteriophage may activate toll-like receptors, leading to aggregated intestinal inflammation and clinical symptoms [34,35]. Because an inflamed TME predisposes tumor growth [20,36], whether this bacteriophage-induced inflammation may worsen CRC requires additional study. When we tested ØTCUFN3 in a healthy mice model, we found that ØTCUFN3 indeed activates the immune response, but this immune response has no, or only minimal, effect on the host (Figure 8). Nevertheless, precautions should always be taken when applying phage therapy in the future.

Although promising, bacteriophage resistance can also arise during phage therapy [37]. It was suggested that bacteria can develop mutations or variations in phage-binding receptors, leading to decreased bacteriophage infection. The bacteria also employed several defense mechanisms against bacteriophage, such as DNA interference, cell suicide, and the production of metabolites that prevent phage DNA entry or replication [37]. Bacteriophage resistance resulting from the use of phage therapy was found clinically [38,39,40]. It has been suggested that the host immune response plays a crucial role in a successful phage therapy, where neutrophils help eliminate phage-resistant bacterial subpopulations [41]. Thereby, an immunocompetent status may be necessary for phage therapy. In addition, phage cocktails may also provide a means for reducing the risk of emergent phage resistance [42].

The experiment here does present several limitations. First, we performed xenograft implantation in immunocompetent mice, which may cause rejection. Although our experiments ended before rejection occurs (on the 8th day post-implantation), as indicated by the lack of immune cell infiltration into the graft tissue (Figure 6a), we cannot rule out the possibility that the observed bacteriophage-induced immune infiltration (Figure 6b,d) was associated with graft rejection. As immunity is one of the essential components of the TME, the intratumoral microbiome interacts with it to affect tumor growth in many aspects. In addition to the targeting effect against *Fusobacterium*, we also wanted to observe the potential immune-regulatory effect of the bacteriophage. Although possible immune involvement was observed within the xenograft tissue, further studies using immunodeficient mice or a syngeneic CRC model may be warranted. In addition, most *F. nucleatum* strains used in this study were obtained from different patient specimen sources. As the isolated ØTCUFN3 were specific to only a few strains within *F. nucleatum*, knowing the characteristics of each bacterial strain may enable us to understand more about the phage. However, due to patients’ confidentiality, we could not identify the sources of each bacteria strain. Future studies performed on bacterial strains with well-known backgrounds and biological and molecular characteristics may help understand the therapeutic potential of ØTCUFN3. In addition, testing the phage on *F. nucleatum* isolated from CRC samples may provide insights into phage therapy against cancers.

## 4. Materials and Methods

### 4.1. Bacteria and Cultivation

Thirty-two *F. nucleatum* clinical isolates were isolated from Hualien Tzu Chi Hospital, Taiwan. One reference strain, BCRC 17679, was collected from the Bioresource Collection and Research Center (BCRC), Hsinchu, Taiwan. All clinical isolates were confirmed as *F. nucleatum* by matrix-assisted laser desorption ionization-time of flight (MALDI-TOF). The bacterial strains used in this study are listed in Table 1. All bacterial cultures were cultured on CDC anaerobe 5% sheep blood agar (BBL, Becton Dickinson Microbiology Systems, Sparks, MD, USA), brain heart infusion (BHI) broth (Liofilchem, Roseto degli Abruzzi, Italy), or BBL Fluid thioglycollate medium (FTM) broth (Liofilchem) and incubated in an anaerobic chamber at 37 °C.

### 4.2. Bacteriophage Isolation and Propagation

Sewage samples were collected from Hualien Tzu Chi Hospital, Taiwan. The samples were centrifuged at 3500× *g* for 10 min, after which the supernatants were filtered through a 0.45 μm filter. Bacteriophage enrichment was carried out by mixing a one-milliliter *F. nucleatum* mixture containing different strains (OD600 = 1.0) with 40 mL of sewage filtrate and incubating it anaerobically at 37 °C for 24 h. The mixtures were centrifuged at 6000× *g* for 10 min, followed by filtration through 0.45 μm filters to eliminate any remaining bacteria. Afterward, bacteriophages were identified by the spot method, which was carried out by spotting 10 μL supernatant onto the bacterial lawns on 1.2% FTM agar and incubated anaerobically at 37 °C for 48 h.

Once a single plaque was confirmed on the agar plate, it was isolated and transferred into a new culture. The process was repeated multiple times to purify a homogeneous bacteriophage. The purified phages were stored at −80 °C in 30% glycerol for subsequent experiments.

### 4.3. Genomic Sequencing and Data Analysis

Bacteriophage DNA was extracted and analyzed by paired-end sequencing with the MiSeq system (Illumina, Inc., San Diego, CA, USA) and the Oxford Nanopore system (Oxford, UK). The sequencing reads were processed using PATRIC (https://www.patricbrc.org, accessed on 20 September 2024). The trimming produced 128,638 pairs of short reads with an average length of 136 bp and 10,109 long reads with an average length of 7656 bp. Short and long reads were assembled using Unicycler version 0.4.8, followed by polishing through Racon version 1.5.0 and Pilon version 1.24. The assembly yielded a single contig with a short-read coverage of 164× and a long-read coverage of 427×. The termini of the phage genome were predicted using PhageTerm version 1.0.12, and putative protein-encoding open reading frames (ORFs) were predicted using Prodigal version 2.6.3. The resultant genome spans 127,354 bp and contains 153 ORFs. Protein functions were annotated by running NCBI protein BLAST (https://www.ncbi.nlm.nih.gov/, accessed on 20 September 2024) against the protein database, limited to the virus organism (taxid 10239). Genome alignment between TCUFN3, Fnu1 (NC_055035), and phiKSUM (OR492276) was performed using Mauve version 2.4.0. Default parameters were used for all software unless otherwise noted. The genomic sequence of phage TCUFN3 was submitted to NCBI under a GenBank accession number PQ464787.

### 4.4. Determination of the Bacteriopahge Host Range

The host range was identified using both the spot method and the double-layer agar method on different *F. nucleatum* strains listed in Table 1. The spot method was carried out by distributing the bacteria evenly on a 1.2% FTM agar plate, followed by spotting 10 μL phage lysate (1 × 10^9^ PFU/mL) onto the bacterial lawns. Plaque was observed after anaerobic incubation at 37 °C for 48 h. For the double-layer agar method, the bacteriophages were 10-fold diluted. Then, 100 μL of diluted phages and 100 μL of 1 × 10^7^ CFU *F. nucleatum* were added into 10 mL of 0.6% BHI soft agar medium. The mixture was then overlaid onto the FTM agar. The plates were incubated anaerobically at 37 °C for 48 h and observed for plaque formation. Based on the result, *F. nucleatum* 34597 was chosen as the optimal host for the phages identified.

### 4.5. Transmission Electron Microscopy (TEM)

Ten microliters of purified phage lysate (1 × 10^9^ PFU/mL) were deposited on a formvar-coated 200-mesh copper grid. The grids were washed and stained with 2% uranyl acetate. The bacteriophage was visualized using a Hitachi H-7500 transmission electron microscope (Hitachi Company, Tokyo, Japan) operated at an acceleration voltage of 80 kV.

### 4.6. Determination of Phage Multiplicity of Infection (MOI)

The multiplicity of infection (MOI) of the bacteriophage was determined by adding bacteriophages at different MOIs (from 0.01 to 100) into the host bacteria *F. nucleatum* 34597 at the early log phase (OD_600_ = 0.5). The bacteriophage-bacteria mixtures were then incubated at 37 °C anaerobically for 48 h. Following centrifugation at 3500× *g* for 10 min, the supernatants were serially diluted, and the phage titer was determined by the double-layer agar method [43].

### 4.7. Infection Assay

Bacteriophages at different MOIs (from 0.01 to 100) were added to the host strain *F. nucleatum* 34597 at OD_600_ = 0.5 and incubated anaerobically at 37 °C. Bacterial growth was monitored over time by measuring OD_600_ at a 4 h interval for 56 h.

### 4.8. Determination of the Phage Stability Against Different Temperatures and pH

To determine how temperature affects ØTCHFN3 stability, 1 mL of phage (10^9^ PFU/mL) was resuspended in SM Buffer (50 mM Tris HCl, 100 mM NaCl, 8 mM MgSO_4_; pH 7.5) and incubated for one hour at six different temperatures (4, 25, 37, 50, 60, 70 °C). Phage titers were determined by the double-layer method. On the other hand, the stability of ØTCHFN3 against pH variations was determined by suspending 1 mL of phage preparations (10^9^ PFU/mL) in SM buffer adjusted at different pH values (pH 2, 4, 7, and 11) and incubated at 25 °C for 1 h. Phage titers were determined by the double-layer method.

### 4.9. Cell Lines and Culture

Two human colorectal cancer cell lines were used in this study: HCT116 p53^+/+^ (ATCC: #CCL-247) was purchased from Bioresource Collection and Research Center (BCRC; Hsinchu, Taiwan), and p53^−/−^ isogenic HCT116 cells were kindly provided by Professor Ren-In You (Tzu Chi University, Taiwan). HCT116 p53^+/+^ were maintained in McCoy’s 5A medium, whereas HCT116 p53^−/−^ were maintained in Roswell Park Memorial Institute (RPMI) 1640 medium. Both mediums were supplemented with 10% fetal bovine serum (FBS; Gibco; Grand Island, NY, USA), 1% L-glutamine (HyClone; Logan, UT, USA), 1% nonessential amino acids (NEAA; HyClone), 1% sodium pyruvate (HyClone), and 1% penicillin-streptomycin (HyClone). All cell lines were maintained at 37 °C in a humidified atmosphere of 5% CO_2_ following standard operating procedures. The genotype of these cell lines is stable as determined periodically by PCR analysis (Figure S3).

### 4.10. Cell Proliferation Assay

Cells were seeded at a density of 1 × 10^3^ cells per well in 96-well plates. After culturing overnight, the cells were treated with different MOIs of *F. nucleatum* and ØTCHFN3 for 24 h. Cell proliferation was subsequently measured via Cell Counting Kit-8 (CCK-8; Cyrusbioscience, Taipei, Taiwan), according to the manufacturer’s instructions. The absorbance was measured at 450 nm.

### 4.11. RNA Extraction and Quantitative Reverse Transcription PCR (qRT-PCR)

Cells were seeded at a density of 8 × 10^5^ cells per well in a 60-mm-diameter dish. Afterwards, the cells were treated with *F. nucleatum* at a MOI of 100 and ØTCHFN3 at a MOI of 10 against bacteria for 24 h. Total RNA of the cells was isolated by TRIzol reagent (Invitrogen; Carlsbad, CA, USA), and five micrograms of RNA were then converted into complementary DNA by GScript First-Strand Synthesis Kit (GeneDireX, New Taipei City, Taiwan). The PCR reaction was carried out using 2× qPCRBIO SyGreen Blue Mix Lo-ROX (PCR Biosystems, London, UK) with the Roche LightCycler 480 system (Roche Diagnostics; Basel, Switzerland). The reaction cycling was performed by an initial denaturation at 95 °C for 2 min and 45 cycles of denaturation (95 °C, 15 s), annealing (58 °C, 25 s), and extension (72 °C, 20 s). Relative gene expression was calculated using the 2^−ΔΔCT^ method, with gene expression levels normalized to the housekeeping gene *β-actin*. The oligonucleotide primers used are shown in Table S2.

### 4.12. Animal Experiments

To study the effect of ØTCHFN3 in *F. nucleatum*-associated CRC, sixteen six-week-old male BALB/c were randomly assigned into four groups, with each group receiving (A) vehicle, (B) 1 × 10^6^ CFU *F. nucleatum*, (C) 1 × 10^7^ PFU ØTCHFN3, and (D) both *F. nucleatum* and ØTCHFN3, respectively. For each 200 μL of sterile PBS, 1 × 10^7^ HCT-116 p53^−/−^ cells were suspended and were injected subcutaneously into the right flanks of these mice. For mice also receiving *F. nucleatum*, 1 × 10^6^ CFU *F. nucleatum* was mixed into the suspended cells right before the injection. On days 3, 5, and 7 post-xenografts, the mice were injected locally (near the xenograft tumor) with 1 × 10^7^ PFU ØTCHFN3 (suspended in 50 μL sterile PBS) or PBS vehicle. Body weight and tumor volume were recorded daily. Tumor volume was measured using the equation: V = π/6 (L × W × H), where “L”, “W”, and “H” represent the length, width, and height of the xenograft, respectively. Mice were sacrificed eight days after injection, and xenograft tumors were excised and weighed.

To investigate the safeness and immune-modulatory effect of ØTCHFN3, another ten mice were assigned into two groups, each receiving an intraperitoneal injection of PBS (vehicle) or 1 × 10^7^ ØTCHFN3 (suspended in 100 μL of sterile PBS) three times a week for four weeks. Mice were weighed every day, blood was drawn every week, and organs were collected on the day of sacrifice for investigation.

### 4.13. Hematoxylin and Eosin (H&E) Staining

Tissues were fixed in 10% formalin for 24 h and processed as described previously [44]. Before staining, the slides were deparaffinized and rehydrated. The rehydrated sections were stained for H&E as follows: hematoxylin (Merck, Darmstadt, Germany), acid alcohol (1% HCl), eosin, 95% ethanol, 100% ethanol, and Sub-X xylene substitute. The histological investigation was performed as previously described [44].

### 4.14. Immunohistochemistry (IHC) Staining

Antigen retrieval was carried out by incubating the rehydrated slides in boiling sodium citrate buffer for 20 min. The slides were then incubated with 3% H_2_O_2_ for 10 min and 10% FBS (Gibco) for 1 h. Subsequently, the slides were stained overnight at 4 °C with a Ki-67 (1:100; Cat# A20018; Abclonal; Wuhan, China) primary antibodies. After washing, the slides were stained with HRP-conjugated secondary antibody (1:1000 dilution; Cat# AS014; ABclonal) for 30 min, followed by visualization with 3, 3′-diaminobenzidine (DAB; Thermo Scientific) for 5 min. Sections were then counterstained with hematoxylin (Merck) and dehydrated with increasing concentrations of ethanol and Sub-X xylene substitute.

### 4.15. Quantification of F. nucleatum in Tumor Tissue

DNA was extracted from the tumor tissue following the manufacturer’s protocol (QIAGEN, Germantown, MD, USA). The absolute abundance of *F. nucleatum* in the DNA was analyzed by qRT-PCR by denaturation at 95 °C for 2 min and 45 cycles of denaturation (95 °C, 5 s), annealing (60 °C, 30 s), and extension (72 °C, 20 s). The CT values obtained were plotted against a standard curve of log 10 concentration of a decimal serial dilution of *F. nucleatum*. The standard curve is assured to be linear over all 6 orders of magnitude with an R^2^ value of 0.996. The oligonucleotide primers used are shown in Table S2.

### 4.16. Measurement for ØTCHFN3-Specific IgG Response and Cytokine Concentrations

To investigate the specific IgG response, 96-well ELISA plates were coated overnight at 4 °C with 100 μL of 20 μg/mL ØTCHFN3. Following blocking with 5% non-fat milk, 100 μL/well serum samples (at 1:800 dilution) were added and incubated for 2 h. The plates were then incubated for 30 min with HRP-conjugated anti-mouse IgG antibody (1:10,000 dilution; Cat# AS003; ABclonal). Visualization was carried out by incubating the plates with 100 μL/well TMB substrate for 15 min in the dark. The reaction was stopped with 1 N hydrochloric acid, and the optical density (OD) was read at 450 nm.

Concentrations of IL-1β and IFN-γ were measured using a commercial sandwich ELISA kit (Cat# 432604 for IL-1β and Cat# 430801 for IFN-γ; BioLegend, San Diego, CA, USA), according to the manufacturer’s protocol.

## 5. Conclusions

Bacteriophages have been an effective strategy to fight multi-drug-resistant bacteria. Current knowledge of the relationship between intratumoral microbiota and tumor formation has led to incorporating bacteriophages in cancer therapy. However, unintended adverse effects may occur during the use of bacteriophages; therefore, treatment strategies containing bacteriophages may require deeper investigation. In addition, the strain diversity within the same bacteria may vary in their susceptibility to bacteriophage treatment and should be taken into consideration in the future.

## Figures and Tables

**Table 1:** Strains of *Fusobacterium nucleatum* used in this study and their host range.

Bacterial Strains	Sources	Spot Test	Plaque Formation
BCRC17679	BCRC	−	−
4674	Hualien Tzu Chi Hosptial, Taiwan	−	−
10681		−	−
16340		−	−
23712		−	−
34597		+	+
38017		−	−
42704		−	−
47167		+	+
66805		−	−
90186		−	−
97747		−	−
124924		−	−
150672		+	−
21658		−	−
8182		+	−
13647		−	−
18729		+	−
28793		−	−
30366		−	−
33548		+	−
38539		−	−
45927		+	−
56629		−	−
64437		−	−
108621		−	−
112173		+	−
131959		+	−
132401		−	−
136804		+	−
52227		−	−
68386		+	−
62793		−	−

BCRC, Bioresource Collection and Research Center, Taiwan; −, no growth; +, growth.

## Data Availability

All the original data presented in the study have been deposited in Mendeley Data (doi:10.17632/hndps6gngr.1). Sequencing data of ØTCUFN3 was deposited to the NCBI GenBank, under an accession number of PQ464787.

## Supplementary Materials

The following supporting information can be downloaded at: https://www.mdpi.com/article/10.3390/antibiotics14010045/s1, Figure S1: (a–c) Genome alignment between (a) TCUFN3, (b) Fnu1 (NC_055035), and (c) phiKSUM (OR492276). The alignment was performed using Mauve version 2.4.0. Protein alignment of the (d) predicted portal protein and (e) predicted terminase of ØTCUFN3 with that of Fnu1 (NC_055035) and phiKSUM (OR492276); Figure S2: Proliferation analysis of HCT116 p53^+/+^ and HCT-116 p53^−/−^ cells treated with *F. nucleatum* and ØTCUFN3. Proliferation of (a,c) HCT116 p53^+/+^ and (b,d) HCT-116 p53^−/−^ cells treated with different MOIs of (a,b) *F. nucleatum* and (c,d) ØTCUFN3. The cells were cultured for 24 h, and proliferation was measured using a CCK-8 assay at a wavelength of 450 nm. Bacterial control and ØTCUFN3 control indicate the background absorbance of the bacteria or phage itself (without cells). Results are presented as the mean ± S.D. from three or four independent experiments. ** *p*-value < 0.01 and *** *p*-value < 0.001; significance according to one-way ANOVA; Figure S3: PCR analysis of the p53 expression in HCT116 p53^+/+^ and HCT-116 p53^−/−^ cells. NC, no template control; Table S1: Functional classification of 153 ORFs in the ØTCUFN3 genome; Table S2: Primer pairs used in this study.
