# Supplementary material for: A Novel Bacteriophage with the Potential to Inhibit Fusobacterium nucleatum-Induced Proliferation of Colorectal Cancer Cells
Source: Antibiotics (Basel). 2025 Jan 7;14(1):45. doi: 10.3390/antibiotics14010045 (PMC11760851; doi:10.3390/antibiotics14010045)
Supplement: Supplementary file 1 [file antibiotics-14-00045-s001.zip › antibiotics-3301443-supplementary.pdf]

antibiotics

MDPI

*Supplementary Materials*

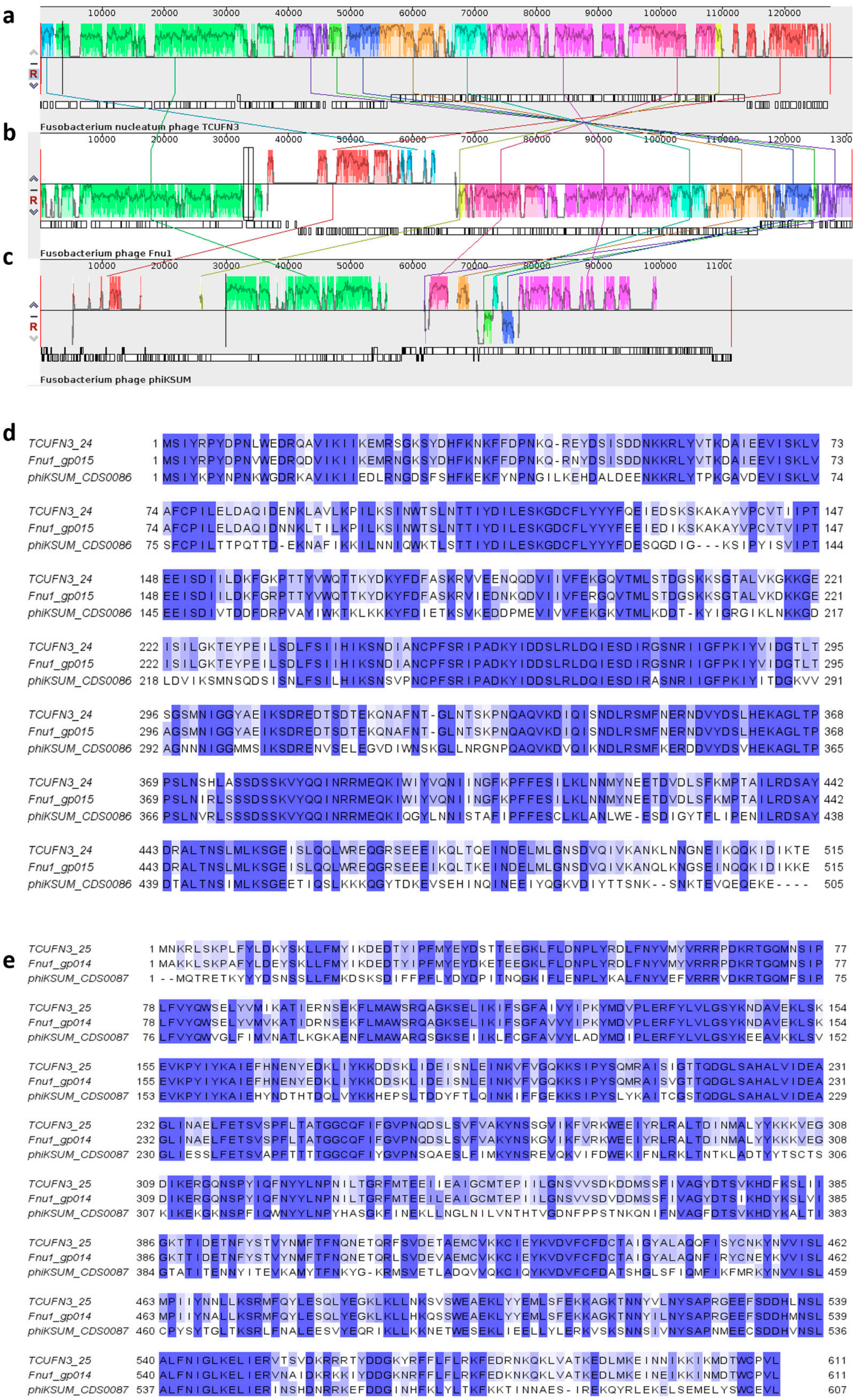


 https://doi.org/10.3390/antibiotics14010045

**Figure S1.** (a–c) Genome alignment between (a) TCUFN3, (b) Fnu1 (NC_055035), and (c) phiKSUM (OR492276). The alignment was performed using Mauve version 2.4.0. Protein alignment of the (d) predicted portal protein and (e) predicted terminase of ØTCUFN3 with that of Fnu1 (NC_055035) and phiKSUM (OR492276).

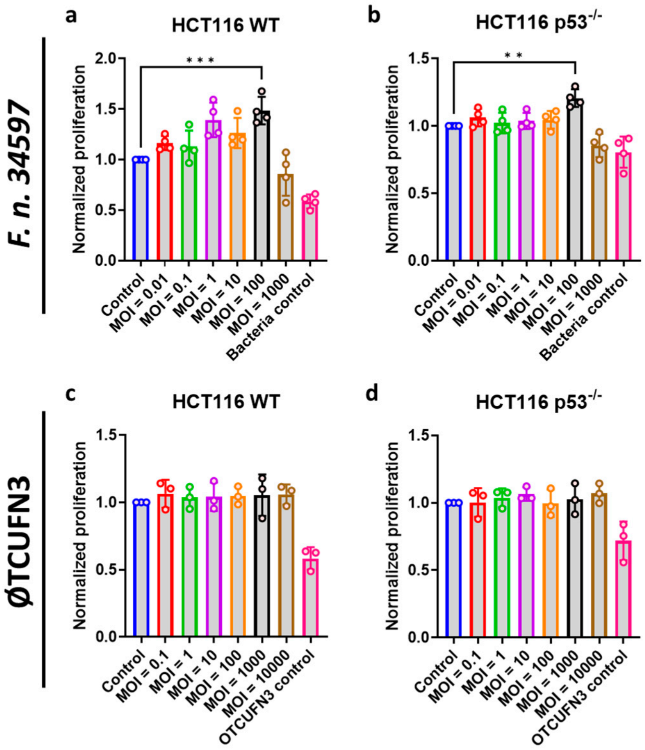


**Figure S2.** Proliferation analysis of HCT116 p53$^{+/+}$ and HCT-116 p53$^{-/-}$ cells treated with *F. nucleatum* and ØTCUFN3. Proliferation of (a,c) HCT116 p53$^{+/+}$ and (b,d) HCT-116 p53$^{-/-}$ cells treated with different MOIs of (a,b) *F. nucleatum* and (c,d) ØTCUFN3. The cells were cultured for 24 h, and proliferation was measured using a CCK-8 assay at a wavelength of 450 nm. Bacterial control and ØTCUFN3 control indicate the background absorbance of the bacteria or phage itself (without cells). Results are presented as the mean ± S.D. from three or four independent experiments. ** $p$-value < 0.01 and *** $p$-value < 0.001; significance according to one-way ANOVA.

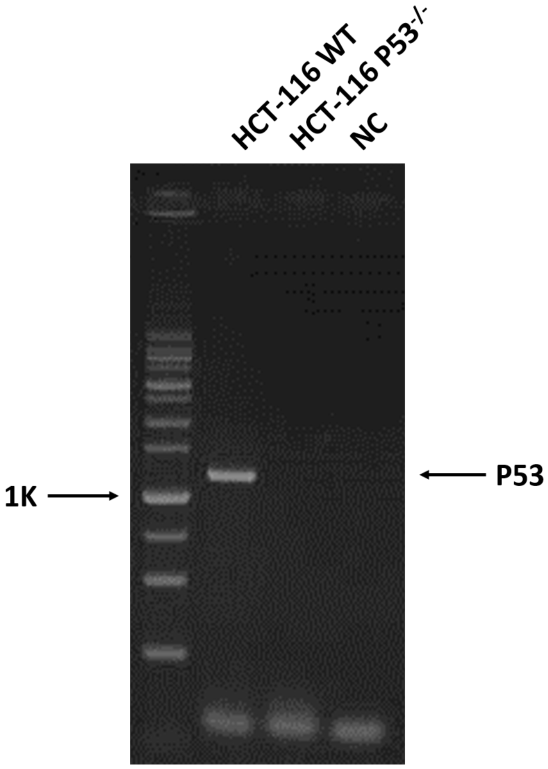


**Figure S3.** PCR analysis of the p53 expression in HCT116 $p53^{+/+}$ and HCT-116 $p53^{-/-}$ cells. NC, no template control.

**Table S1.** Functional classification of 153 ORFs in the ØTCUFN3 genome.

| query acc.ver | subject acc.ver | % identity | alignment length | q. start | q. End | s. start | s. end | evalue | % positives | Description |
|---|---|---|---|---|---|---|---|---|---|---|
| FN3_1 | | | | | | | | | | Hypothetical protein |
| FN3_2 | YP_010082872.1 | 63.454 | 249 | 1 | 248 | 1 | 249 | 1.41E-103 | 80.72 | hypothetical protein KMD24_gp070 [Fusobacterium phage Fnu1] |
| FN3_3 | YP_010082882.1 | 90.323 | 155 | 1 | 155 | 1 | 155 | 2.23E-91 | 95.48 | molecular chaperone [Fusobacterium phage Fnu1] |
| FN3_4 | YP_010082833.1 | 93.642 | 346 | 1 | 346 | 954 | 1299 | 0 | 97.98 | DUF1983 domain-containing protein [Fusobacterium phage Fnu1] |
| FN3_5 | DAY41500.1 | 89.101 | 367 | 1 | 367 | 1 | 367 | 0 | 93.46 | TPA: endonuclease [Ackermannviridae sp.] |
| FN3_6 | YP_010082833.1 | 96.013 | 928 | 1 | 928 | 1 | 928 | 0 | 98.81 | DUF1983 domain-containing protein [Fusobacterium phage Fnu1] |
| FN3_7 | YP_010082832.1 | 94.355 | 124 | 1 | 124 | 1 | 124 | 3.60E-79 | 97.58 | hypothetical protein KMD24_gp030 [Fusobacterium phage Fnu1] |
| FN3_8 | YP_010082977.1 | 44.444 | 234 | 113 | 339 | 16 | 242 | 1.47E-40 | 64.53 | anti-repressor [Fusobacterium phage Fnu1] |
| FN3_9 | YP_010082830.1 | 89.645 | 338 | 1 | 338 | 1 | 338 | 0 | 95.27 | hypothetical protein KMD24_gp028 [Fusobacterium phage Fnu1] |
| FN3_10 | DAW75187.1 | 60.366 | 1693 | 1 | 1646 | 307 | 1965 | 0 | 75.19 | TPA: minor tail protein [Bacteriophage sp.] |
| FN3_11 | | 41.964 | 112 | 5 | 110 | 7 | 118 | 3.69E-12 | 54.46 | TPA: intron associated endonuclease [Caudoviricetes sp.] |
| FN3_12 | DAW82231.1 | 57.388 | 291 | 1 | 288 | 1 | 291 | 5.34E-105 | 74.91 | TPA: minor tail protein [Bacteriophage sp.] |
| FN3_13 | YP_010082828.1 | 85.047 | 214 | 1 | 214 | 1 | 214 | 9.74E-130 | 95.79 | hypothetical protein KMD24_gp026 [Fusobacterium phage Fnu1] |

| | | | | | | | | | | |
|---|---|---|---|---|---|---|---|---|---|---|
| FN3_14 | YP_010082827.1 | 87.755 | 245 | 1 | 245 | 1 | 245 | 1.71E-146 | 96.73 | hypothetical protein KMD24_gp025 [Fusobacterium phage Fnu1] |
| FN3_15 | DAW75200.1 | 91.71 | 386 | 3 | 387 | 4 | 385 | 0 | 94.56 | TPA: Poxvirus A32 protein [Bacteriophage sp.] |
| FN3_16 | YP_010082825.1 | 81.991 | 211 | 1 | 211 | 1 | 211 | 6.08E-122 | 92.42 | hypothetical protein KMD24_gp023 [Fusobacterium phage Fnu1] |
| FN3_17 | DAE26594.1 | 93.889 | 180 | 1 | 180 | 1 | 180 | 7.44E-123 | 98.33 | TPA: hypothetical protein [Ackermannviridae sp. ctaCq7] |
| FN3_18 | DAE26595.1 | 95.935 | 123 | 1 | 123 | 1 | 123 | 5.22E-81 | 97.56 | TPA: hypothetical protein [Ackermannviridae sp. ctaCq7] |
| FN3_19 | DAE26596.1 | 96.97 | 99 | 1 | 99 | 1 | 99 | 2.38E-63 | 100 | TPA: hypothetical protein [Ackermannviridae sp. ctaCq7] |
| FN3_20 | | | | | | | | | | Hypothetical protein |
| FN3_21 | YP_010082820.1 | 82.124 | 386 | 1 | 384 | 1 | 385 | 0 | 90.41 | hypothetical protein KMD24_gp018 [Fusobacterium phage Fnu1] |
| FN3_22 | YP_010082819.1 | 95.425 | 153 | 1 | 153 | 1 | 153 | 1.10E-102 | 98.04 | methyl-CpG-binding domain-containing protein 9 [Fusobacterium.. |
| FN3_23 | YP_010082818.1 | 92.045 | 264 | 1 | 264 | 1 | 263 | 6.35E-156 | 96.21 | hypothetical protein KMD24_gp016 [Fusobacterium phage Fnu1] |
| FN3_24 | YP_010082817.1 | 94.951 | 515 | 1 | 515 | 1 | 515 | 0 | 97.86 | hypothetical protein KMD24_gp015 [Fusobacterium phage Fnu1] |
| FN3_25 | DAE26577.1 | 95.417 | 611 | 1 | 611 | 1 | 611 | 0 | 97.71 | TPA: terminase [Ackermannviridae sp. ctaCq7] |
| FN3_26 | YP_010082815.1 | 96.25 | 240 | 1 | 240 | 1 | 240 | 3.00E-165 | 97.5 | hypothetical protein KMD24_gp013 [Fusobacterium phage Fnu1] |
| FN3_27 | DAX46451.1 | 91.979 | 187 | 1 | 187 | 1 | 187 | 2.40E-114 | 96.79 | TPA: hypothetical protein [Ackermannviridae sp.] |
| FN3_28 | WGH50247.1 | 94.118 | 119 | 1 | 119 | 74 | 192 | 2.13E-72 | 97.48 | hypothetical protein FNU2_120 [Fusobacterium phage vB_FnuS_FNU2 |
| FN3_29 | DAX46283.1 | 99.632 | 272 | 1 | 272 | 1 | 272 | 0 | 100 | TPA: antirepressor protein [Ackermannviridae sp.] |
| FN3_30 | YP_010082812.1 | 53.333 | 225 | 4 | 219 | 11 | 224 | 2.01E-71 | 68.44 | Rha-like transcriptional regulator [Fusobacterium phage Fnu1] |
| FN3_31 | DAX66608.1 | 97.101 | 69 | 1 | 69 | 1 | 69 | 2.24E-40 | 98.55 | TPA: Ribbon-helix-helix domain [Bacteriophage sp.] |
| FN3_32 | DAX46324.1 | 97.647 | 255 | 1 | 255 | 1 | 255 | 0 | 99.22 | TPA: adenine-specific methyltransferase [Ackermannviridae sp.] |
| FN3_33 | YP_010082810.1 | 92.174 | 115 | 1 | 115 | 24 | 138 | 2.29E-71 | 97.39 | ComF family protein [Fusobacterium phage Fnu1] |
| FN3_34 | YP_010082809.1 | 93.416 | 243 | 1 | 243 | 1 | 243 | 1.20E-160 | 97.12 | transposase [Fusobacterium phage Fnu1] |
| FN3_35 | YP_010082808.1 | 89.091 | 275 | 1 | 275 | 1 | 275 | 4.89E-169 | 95.64 | nucleotidyltransferase [Fusobacterium phage Fnu1] |
| FN3_36 | YP_010082806.1 | 85.496 | 131 | 1 | 131 | 1 | 131 | 5.53E-64 | 94.66 | hybrid sensor histidine kinase/response regulator [Fusobacteri. |
| FN3_37 | YP_010082805.1 | 88.333 | 60 | 1 | 60 | 10 | 69 | 9.75E-33 | 98.33 | hypothetical protein KMD24_gp003 [Fusobacterium phage Fnu1] |
| FN3_38 | | | | | | | | | | Hypothetical protein |
| FN3_39 | DAP06232.1 | 31.004 | 229 | 484 | 645 | 77 | 295 | 5.49E-14 | 47.16 | tail fiber protein [Fusobacterium phage Fnu1] |
| FN3_40 | YP_010082983.1 | 87.879 | 99 | 1 | 99 | 1 | 99 | 3.81E-58 | 93.94 | hypothetical protein KMD24_gp181 [Fusobacterium phage Fnu1] |
| FN3_41 | YP_010082982.1 | 95.135 | 185 | 1 | 185 | 1 | 185 | 7.71E-126 | 97.84 | endolysin [Fusobacterium phage Fnu1] |
| FN3_42 | DAP06230.1 | 95.652 | 138 | 1 | 138 | 1 | 138 | 2.55E-92 | 99.28 | TPA: holin [Ackermannviridae sp.] |
| FN3_43 | YP_010082980.1 | 96.552 | 116 | 1 | 116 | 1 | 116 | 2.33E-77 | 98.28 | tail assembly chaperone [Fusobacterium phage Fnu1] |

| | | | | | | | | | | |
|---|---|---|---|---|---|---|---|---|---|---|
| FN3_44 | YP_010082979.1 | 87.952 | 249 | 1 | 249 | 1 | 249 | 7.33E-159 | 95.18 | hypothetical protein KMD24_gp177 [Fusobacterium phage Fnu1] |
| FN3_45 | DAP76659.1 | 49.416 | 257 | 6 | 250 | 2 | 254 | 1.46E-76 | 67.32 | TPA: KilAC domain protein [Caudoviricetes sp.] |
| FN3_46 | | | | | | | | | | Hypothetical protein |
| FN3_47 | YP_010082976.1 | 89.583 | 96 | 1 | 96 | 1 | 95 | 5.13E-52 | 94.79 | DNA binding protein [Fusobacterium phage Fnu1] |
| FN3_48 | YP_010082974.1 | 85.526 | 76 | 34 | 109 | 1 | 76 | 7.95E-41 | 94.74 | glutaredoxin [Fusobacterium phage Fnu1] |
| FN3_49 | DAM61809.1 | 89.669 | 242 | 1 | 242 | 1 | 242 | 6.25E-161 | 95.87 | TPA: adenine-specific methyltransferase [Ackermannviridae sp.] |
| FN3_50 | DAE26584.1 | 92.766 | 235 | 1 | 235 | 1 | 235 | 5.12E-158 | 97.45 | TPA: adenine-specific methyltransferase [Ackermannviridae sp... |
| FN3_51 | YP_010082964.1 | 42.857 | 287 | 5 | 290 | 117 | 379 | 1.06E-62 | 65.16 | anti-repressor [Fusobacterium phage Fnu1] |
| FN3_52 | YP_010082971.1 | 90.191 | 734 | 1 | 732 | 1 | 733 | 0 | 94.82 | RNA ligase [Fusobacterium phage Fnu1] |
| FN3_53 | YP_010082970.1 | 88.793 | 116 | 1 | 116 | 1 | 116 | 1.45E-64 | 93.1 | TonB-dependent receptor [Fusobacterium phage Fnu1] |
| FN3_54 | YP_010082969.1 | 70.711 | 239 | 1 | 239 | 1 | 239 | 2.60E-121 | 87.87 | hypothetical protein KMD24_gp167 [Fusobacterium phage Fnu1] |
| FN3_55 | YP_010082968.1 | 81.818 | 143 | 1 | 143 | 1 | 143 | 3.89E-80 | 93.71 | hypothetical protein KMD24_gp166 [Fusobacterium phage Fnu1] |
| FN3_56 | YP_010082967.1 | 68.293 | 123 | 1 | 123 | 1 | 123 | 1.20E-53 | 83.74 | hypothetical protein KMD24_gp165 [Fusobacterium phage Fnu1] |
| FN3_57 | YP_010082964.1 | 76.882 | 372 | 1 | 372 | 8 | 379 | 0 | 89.52 | anti-repressor [Fusobacterium phage Fnu1] |
| FN3_58 | YP_010082961.1 | 76.259 | 139 | 3 | 141 | 2 | 139 | 1.37E-72 | 87.77 | heparinase [Fusobacterium phage Fnu1] |
| FN3_59 | YP_010082959.1 | 65.116 | 172 | 1 | 169 | 1 | 172 | 4.73E-73 | 79.65 | hypothetical protein KMD24_gp157 [Fusobacterium phage Fnu1] |
| FN3_60 | YP_010082957.1 | 93.061 | 490 | 1 | 490 | 1 | 489 | 0 | 96.53 | hypothetical protein KMD24_gp155 [Fusobacterium phage Fnu1] |
| FN3_61 | YP_010082956.1 | 95.276 | 254 | 1 | 254 | 1 | 254 | 6.56E-172 | 97.64 | exonuclease V [Fusobacterium phage Fnu1] |
| FN3_62 | YP_010082956.1 | 87.44 | 414 | 1 | 414 | 267 | 677 | 0 | 94.44 | exonuclease V [Fusobacterium phage Fnu1] |
| FN3_63 | DAK84672.1 | 33.333 | 63 | 62 | 124 | 70 | 132 | 0.026 | 55.56 | TPA: hypothetical protein [Caudoviricetes sp.] |
| FN3_64 | WPH65712.1 | 39.803 | 304 | 2 | 303 | 4 | 290 | 8.38E-61 | 61.51 | anti-repressor [Fusobacterium phage phiKSUM] |
| FN3_65 | YP_010082954.1 | 53.804 | 184 | 1 | 183 | 18 | 200 | 1.86E-64 | 73.91 | hypothetical protein KMD24_gp152 [Fusobacterium phage Fnu1] |
| FN3_66 | YP_010082953.1 | 67.424 | 264 | 1 | 263 | 15 | 277 | 1.12E-134 | 82.95 | tetrahydrofolate dehydrogenase and cyclohydrolase [Fusobacteri. |
| FN3_67 | YP_010082952.1 | 60.355 | 169 | 1 | 162 | 1 | 169 | 7.19E-60 | 78.7 | hypothetical protein KMD24_gp150 [Fusobacterium phage Fnu1] |
| FN3_68 | DAY29667.1 | 43.716 | 183 | 4 | 181 | 3 | 183 | 4.07E-33 | 56.83 | TPA: antirepressor protein [Caudoviricetes sp.] |
| FN3_69 | YP_010082950.1 | 87.5 | 168 | 1 | 168 | 1 | 168 | 5.29E-92 | 94.05 | hypothetical protein KMD24_gp148 [Fusobacterium phage Fnu1] |
| FN3_70 | YP_010082949.1 | 77.869 | 122 | 1 | 122 | 1 | 122 | 7.48E-64 | 90.16 | tRNA-His guanylyltransferase [Fusobacterium phage Fnu1] |
| FN3_71 | DAY19290.1 | 50.125 | 401 | 1 | 398 | 1 | 386 | 7.96E-121 | 68.08 | TPA: endonuclease [Caudoviricetes sp.] |
| FN3_72 | DAX46311.1 | 95.911 | 269 | 1 | 269 | 1 | 269 | 0 | 99.26 | TPA: High frequency of lysogenization C protein... |
| FN3_73 | YP_010082945.1 | 92.308 | 52 | 1 | 52 | 1 | 52 | 5.97E-25 | 98.08 | hypothetical protein KMD24_gp143 [Fusobacterium phage Fnu1] |

| | | | | | | | | | | |
|---|---|---|---|---|---|---|---|---|---|---|
| FN3_74 | YP_010082944.1 | 70.909 | 220 | 1 | 220 | 1 | 219 | 5.87E-108 | 87.27 | TetR family transcriptional regulator [Fusobacterium phage Fnu1 |
| FN3_75 | YP_010082941.1 | 58.125 | 160 | 21 | 178 | 8 | 166 | 1.59E-49 | 71.25 | hypothetical protein KMD24_gp139 [Fusobacterium phage Fnu1] |
| FN3_76 | YP_010082940.1 | 57.831 | 83 | 3 | 85 | 2 | 84 | 1.59E-23 | 80.72 | hypothetical protein KMD24_gp138 [Fusobacterium phage Fnu1] |
| FN3_77 | YP_010082939.1 | 58.824 | 85 | 1 | 85 | 1 | 85 | 5.79E-26 | 71.76 | hypothetical protein KMD24_gp137 [Fusobacterium phage Fnu1] |
| FN3_78 | YP_010082937.1 | 94.527 | 201 | 1 | 201 | 1 | 201 | 3.48E-139 | 99 | ribonuclease HI [Fusobacterium phage Fnu1] |
| FN3_79 | YP_010082936.1 | 79.537 | 259 | 1 | 258 | 1 | 259 | 2.25E-150 | 89.58 | DNA adenine methylase [Fusobacterium phage Fnu1] |
| FN3_80 | YP_010082935.1 | 44.186 | 129 | 1 | 127 | 4 | 132 | 1.44E-26 | 66.67 | hypothetical protein KMD24_gp133 [Fusobacterium phage Fnu1] |
| FN3_81 | YP_010082934.1 | 80.702 | 171 | 1 | 171 | 2 | 172 | 1.76E-95 | 89.47 | hypothetical protein KMD24_gp132 [Fusobacterium phage Fnu1] |
| FN3_82 | DAX46355.1 | 71.277 | 94 | 1 | 93 | 1 | 94 | 2.52E-38 | 88.3 | TPA: hypothetical protein [Ackermannviridae sp.] |
| FN3_83 | DAX46312.1 | 98.707 | 232 | 1 | 232 | 18 | 249 | 6.76E-168 | 100 | TPA: AAA domain protein [Ackermannviridae sp.] |
| FN3_84 | YP_010082932.1 | 90.345 | 145 | 1 | 145 | 1 | 145 | 5.46E-86 | 93.1 | antirepressor [Fusobacterium phage Fnu1] |
| FN3_85 | YP_010082931.1 | 86.453 | 406 | 2 | 392 | 3 | 408 | 0 | 91.13 | hypothetical protein KMD24_gp129 [Fusobacterium phage Fnu1] |
| FN3_86 | YP_010082930.1 | 95.62 | 137 | 1 | 137 | 5 | 141 | 2.82E-87 | 97.81 | polysaccharide biosynthesis protein [Fusobacterium phage Fnu1] |
| FN3_87 | YP_010082929.1 | 89.452 | 493 | 1 | 493 | 1 | 493 | 0 | 95.54 | DNA helicase [Fusobacterium phage Fnu1] |
| FN3_88 | YP_010082928.1 | 90.09 | 333 | 1 | 333 | 1 | 333 | 0 | 94.89 | DNA primase [Fusobacterium phage Fnu1] |
| FN3_89 | YP_010082927.1 | 78.03 | 132 | 124 | 252 | 138 | 262 | 9.74E-60 | 87.12 | anti-repressor [Fusobacterium phage Fnu1] |
| FN3_90 | YP_010082926.1 | 95.755 | 424 | 1 | 424 | 1 | 424 | 0 | 97.17 | exonuclease [Fusobacterium phage Fnu1] |
| FN3_91 | DAW49784.1 | 89.091 | 110 | 1 | 110 | 20 | 129 | 1.02E-64 | 93.64 | TPA: phosphoadenosine-phosphosulfate reductase [Caudoviricetes. |
| FN3_92 | YP_010082925.1 | 88.166 | 169 | 89 | 257 | 1 | 169 | 2.45E-106 | 94.08 | dUTP diphosphatase [Fusobacterium phage Fnu1] |
| FN3_93 | | | | | | | | | | Hypothetical protein |
| FN3_94 | DAO81042.1 | 41.406 | 256 | 8 | 261 | 15 | 253 | 2.97E-49 | 60.16 | TPA: antirepressor [Caudoviricetes sp.] |
| FN3_95 | DAU65660.1 | 96.203 | 474 | 1 | 474 | 1 | 474 | 0 | 98.1 | TPA: DNA polymerase II small subunit [Ackermannviridae sp.] |
| FN3_96 | YP_010082923.1 | 92.825 | 223 | 19 | 241 | 16 | 238 | 4.32E-148 | 97.31 | hypothetical protein KMD24_gp121 [Fusobacterium phage Fnu1] |
| FN3_97 | DAU65664.1 | 95.794 | 214 | 1 | 214 | 1 | 214 | 5.67E-148 | 98.6 | TPA: Lysozyme [Ackermannviridae sp.] |
| FN3_98 | YP_010082920.1 | 93.125 | 160 | 1 | 160 | 1 | 160 | 7.28E-107 | 98.12 | DNA repair protein MmcB-related protein [Fusobacterium phage... |
| FN3_99 | YP_010082918.1 | 61.722 | 209 | 1 | 208 | 1 | 205 | 1.65E-75 | 77.51 | VP1 protein [Fusobacterium phage Fnu1] |
| FN3_100 | WGH50198.1 | 78.454 | 427 | 1 | 427 | 1 | 427 | 0 | 86.42 | putative ATP dependent DNA ligase domain protein [Fusobacteriu. |
| FN3_101 | YP_010082916.1 | 89.024 | 82 | 11 | 92 | 1 | 82 | 1.49E-45 | 96.34 | hypothetical protein KMD24_gp114 [Fusobacterium phage Fnu1] |
| FN3_102 | YP_010082915.1 | 100 | 29 | 1 | 29 | 1 | 29 | 8.77E-11 | 100 | isovaleryl-CoA dehydrogenase [Fusobacterium phage Fnu1] |
| FN3_103 | DAL24016.1 | 40.972 | 432 | 4 | 430 | 7 | 404 | 7.33E-83 | 59.03 | TPA: endonuclease [Caudoviricetes sp.] |

| | | | | | | | | | | |
|---|---|---|---|---|---|---|---|---|---|---|
| FN3_104 | YP_010082914.1 | 88.155 | 515 | 1 | 514 | 4 | 518 | 0 | 93.2 | RNA-binding protein [Fusobacterium phage Fnu1] |
| FN3_105 | YP_010082913.1 | 59.677 | 62 | 1 | 62 | 1 | 62 | 2.05E-13 | 69.35 | hypothetical protein KMD24_gp111 [Fusobacterium phage Fnu1] |
| FN3_106 | YP_010082955.1 | 72.832 | 173 | 10 | 182 | 13 | 185 | 9.11E-92 | 87.86 | DNA methyltransferase [Fusobacterium phage Fnu1] |
| FN3_107 | DAO30960.1 | 93.5 | 200 | 1 | 200 | 1 | 200 | 1.60E-139 | 98 | TPA: Thymidylate synthase thyX [Ackermannviridae sp.] |
| FN3_108 | DAQ26560.1 | 77.372 | 137 | 110 | 246 | 118 | 251 | 1.11E-68 | 88.32 | TPA: antirepressor protein [Ackermannviridae sp.] |
| FN3_109 | YP_010082910.1 | 89.756 | 205 | 1 | 205 | 1 | 205 | 5.28E-124 | 98.05 | hypothetical protein KMD24_gp108 [Fusobacterium phage Fnu1] |
| FN3_110 | YP_010082908.1 | 82.517 | 143 | 1 | 143 | 1 | 143 | 1.34E-83 | 90.91 | phosphatase [Fusobacterium phage Fnu1] |
| FN3_111 | YP_010082907.1 | 87.395 | 119 | 1 | 119 | 1 | 119 | 1.71E-69 | 93.28 | hypothetical protein KMD24_gp105 [Fusobacterium phage Fnu1] |
| FN3_112 | YP_010082906.1 | 90.909 | 143 | 4 | 146 | 15 | 157 | 1.35E-90 | 97.2 | hypothetical protein KMD24_gp104 [Fusobacterium phage Fnu1] |
| FN3_113 | YP_010082905.1 | 97.126 | 174 | 1 | 174 | 1 | 174 | 2.31E-117 | 99.43 | Mu Gam-like end protection [Fusobacterium phage Fnu1] |
| FN3_114 | YP_010082904.1 | 84.286 | 140 | 1 | 140 | 1 | 139 | 2.97E-75 | 90.71 | Holliday junction resolvase [Fusobacterium phage Fnu1] |
| FN3_115 | YP_010082903.1 | 93.548 | 279 | 1 | 279 | 9 | 287 | 0 | 98.92 | Fic family protein [Fusobacterium phage Fnu1] |
| FN3_116 | YP_010082901.1 | 87.85 | 107 | 1 | 107 | 5 | 111 | 2.46E-62 | 94.39 | hypothetical protein KMD24_gp099 [Fusobacterium phage Fnu1] |
| FN3_117 | YP_010082900.1 | 75.102 | 245 | 1 | 245 | 43 | 287 | 3.73E-136 | 89.39 | guanylate kinase [Fusobacterium phage Fnu1] |
| FN3_118 | YP_010082899.1 | 95.594 | 749 | 1 | 749 | 72 | 820 | 0 | 98.13 | DNA polymerase [Fusobacterium phage Fnu1] |
| FN3_119 | DAQ26592.1 | 90.511 | 137 | 1 | 137 | 1 | 137 | 6.34E-74 | 98.54 | TPA: hypothetical protein [Ackermannviridae sp.] |
| FN3_120 | WGH50325.1 | 72.5 | 40 | 1 | 40 | 1 | 40 | 1.04E-05 | 92.5 | hypothetical protein FNU2_18 [Fusobacterium phage vB_FnuS_FNU2] |
| FN3_121 | YP_010082897.1 | 47.841 | 301 | 1 | 301 | 1 | 299 | 4.04E-93 | 68.11 | hypothetical protein KMD24_gp095 [Fusobacterium phage Fnu1] |
| FN3_122 | DAX46384.1 | 96.788 | 467 | 1 | 467 | 1 | 467 | 0 | 99.14 | TPA: Cas system-associated protein [Ackermannviridae sp.] |
| FN3_123 | YP_010082896.1 | 64.835 | 91 | 1 | 90 | 20 | 110 | 3.09E-37 | 86.81 | tyrosine recombinase [Fusobacterium phage Fnu1] |
| FN3_124 | YP_010082895.1 | 63.333 | 60 | 1 | 60 | 3 | 62 | 1.15E-18 | 73.33 | hypothetical protein KMD24_gp093 [Fusobacterium phage Fnu1] |
| FN3_125 | YP_010082894.1 | 88.811 | 143 | 1 | 143 | 1 | 143 | 2.82E-91 | 97.9 | guanylate-binding protein 6-like protein [Fusobacterium phage.. |
| FN3_126 | YP_010082893.1 | 66.667 | 120 | 1 | 119 | 1 | 117 | 9.01E-44 | 80.83 | hypothetical protein KMD24_gp091 [Fusobacterium phage Fnu1] |
| FN3_127 | YP_010082892.1 | 65.587 | 247 | 3 | 249 | 1 | 246 | 2.64E-108 | 81.38 | hypothetical protein KMD24_gp090 [Fusobacterium phage Fnu1] |
| FN3_128 | DAM61881.1 | 83.333 | 36 | 1 | 36 | 1 | 36 | 2.24E-14 | 94.44 | TPA: Tetrahydromethanopterin S-methyltransferase subunit A... |
| FN3_129 | YP_010082888.1 | 50.641 | 156 | 1 | 156 | 5 | 159 | 2.21E-40 | 67.31 | hypothetical protein KMD24_gp086 [Fusobacterium phage Fnu1] |
| FN3_130 | YP_010082887.1 | 75.61 | 369 | 1 | 369 | 1 | 365 | 1.21E-171 | 86.99 | hypothetical protein KMD24_gp085 [Fusobacterium phage Fnu1] |
| FN3_131 | YP_010082837.1 | 73.18 | 261 | 1 | 257 | 1 | 261 | 6.88E-139 | 85.06 | hypothetical protein KMD24_gp035 [Fusobacterium phage Fnu1] |
| FN3_132 | YP_010082836.1 | 70.062 | 324 | 1 | 323 | 1 | 319 | 7.04E-162 | 83.33 | hypothetical protein KMD24_gp034 [Fusobacterium phage Fnu1] |

| | | | | | | | | | | |
|---|---|---|---|---|---|---|---|---|---|---|
| FN3_133 | YP_010082847.1 | 67.969 | 128 | 1 | 128 | 1 | 124 | 5.03E-50 | 77.34 | hypothetical protein KMD24_gp045 [Fusobacterium phage Fnu1] |
| FN3_134 | YP_010082848.1 | 58.763 | 97 | 1 | 97 | 1 | 97 | 5.66E-31 | 74.23 | hypothetical protein KMD24_gp046 [Fusobacterium phage Fnu1] |
| FN3_135 | DAX46346.1 | 40.741 | 216 | 1 | 209 | 1 | 216 | 1.13E-35 | 61.57 | TPA: hypothetical protein [Ackermannviridae sp.] |
| FN3_136 | | | | | | | | | | Hypothetical protein |
| FN3_137 | | | | | | | | | | Hypothetical protein |
| FN3_138 | DAX46390.1 | 76.316 | 76 | 1 | 76 | 1 | 76 | 7.08E-32 | 90.79 | TPA: hypothetical protein [Ackermannviridae sp.] |
| FN3_139 | YP_010082850.1 | 72.603 | 73 | 39 | 107 | 31 | 103 | 1.03E-24 | 79.45 | hypothetical protein KMD24_gp048 [Fusobacterium phage Fnu1] |
| FN3_140 | DAO66588.1 | 54.348 | 92 | 2 | 93 | 17 | 106 | 4.10E-26 | 70.65 | TPA: hypothetical protein [Caudoviricetes sp.] |
| FN3_141 | YP_010082855.1 | 65.649 | 131 | 6 | 136 | 31 | 160 | 3.47E-53 | 78.63 | anaerobic ribonucleotide reductase small subunit [Fusobacteriu. |
| FN3_142 | YP_010082857.1 | 85.185 | 108 | 1 | 108 | 1 | 108 | 3.66E-61 | 92.59 | hypothetical protein KMD24_gp055 [Fusobacterium phage Fnu1] |
| FN3_143 | YP_010082858.1 | 76.821 | 151 | 2 | 152 | 3 | 153 | 3.87E-78 | 88.08 | hypothetical protein KMD24_gp056 [Fusobacterium phage Fnu1] |
| FN3_144 | YP_010082859.1 | 46.259 | 147 | 10 | 156 | 13 | 158 | 1.05E-38 | 66.67 | hypothetical protein KMD24_gp057 [Fusobacterium phage Fnu1] |
| FN3_145 | YP_010082860.1 | 93.003 | 343 | 2 | 344 | 101 | 443 | 0 | 98.25 | anaerobic ribonucleoside reductase large subunit [Fusobacteriu. |
| FN3_146 | YP_010082860.1 | 94 | 100 | 1 | 100 | 1 | 100 | 1.09E-60 | 97 | anaerobic ribonucleoside reductase large subunit [Fusobacteriu. |
| FN3_147 | YP_010082862.1 | 91.667 | 96 | 2 | 97 | 207 | 302 | 1.37E-55 | 95.83 | anaerobic ribonucleoside-triphosphate reductase [Fusobacterium. |
| FN3_148 | YP_010082862.1 | 92.788 | 208 | 1 | 208 | 1 | 208 | 2.31E-140 | 97.12 | anaerobic ribonucleoside-triphosphate reductase [Fusobacterium. |
| FN3_149 | YP_010082864.1 | 88.785 | 107 | 1 | 107 | 1 | 107 | 1.71E-63 | 99.07 | hypothetical protein KMD24_gp062 [Fusobacterium phage Fnu1] |
| FN3_150 | YP_010082865.1 | 92.903 | 155 | 1 | 155 | 1 | 155 | 6.88E-102 | 97.42 | hypothetical protein KMD24_gp063 [Fusobacterium phage Fnu1] |
| FN3_151 | | | | | | | | | | Hypothetical protein |
| FN3_152 | YP_010082866.1 | 52.113 | 142 | 1 | 141 | 4 | 145 | 6.79E-43 | 76.76 | hypothetical protein KMD24_gp064 [Fusobacterium phage Fnu1] |
| FN3_153 | DAX46422.1 | 61.991 | 221 | 1 | 221 | 1 | 221 | 1.69E-97 | 81.45 | TPA: hypothetical protein [Ackermannviridae sp.] |

TPA: A database designed to capture experimental or inferential results that support submitter-provided annotation for, or assembly of, sequence data that the submitter did not directly determine but derived from GenBank primary data.

**Table S2.** Primer pairs used in this study.

| Gene name | Primer pairs (5' - 3') |
|---|---|
| p53 | Forward TCACCATGGAGGAGCCGCAGTCAG<br>Reverse TCAGTCTGAGTCAGGCCCTTCTG |
| E-cadherin | Forward CGAGAGCTACACGTTCACGG<br>Reverse GGGTGTCGAGGGAAAAATAGG |
| N-cadherin | Forward TTTGATGGAGGTCTCCTAACACC<br>Reverse ACGTTTAACACGTTGGAAATGTG |
| Vimentin | Forward GGCTCAGATTCAGGAACAGC<br>Reverse GCTTCAACGGCAAAGTTCTC |
| Snail | Forward TTCTTCTGCGCTACTGCTGCG<br>Reverse GGGCAGGTATGGAGAGGAAGA |
| GAPDH | Forward TCAAGGCTGAGAACGGGAAG<br>Reverse CGCCCCACTTGATTTTGGAG |
| Fusobacterium nucleatum | Forward CWAACGCGATAAGTAATC<br>Reverse TGGTAACATACGAWAGGG |
| IL-1β | Forward GAAATGCCACCTTTTGACAGTG<br>Reverse TGGATGCTCTCATCAGGACAG |
| IFN-γ | Forward GCCACGGCACAGTCATTGA<br>Reverse TGCTGATGGCCTGATTGTCTT |
